# Insights from a chum salmon (*Oncorhynchus keta*) genome assembly regarding whole-genome duplication and nucleotide variation influencing gene function

**DOI:** 10.1093/g3journal/jkad127

**Published:** 2023-06-09

**Authors:** Eric B Rondeau, Kris A Christensen, Hollie A Johnson, Dionne Sakhrani, Carlo A Biagi, Mike Wetklo, Cody A Despins, Rosalind A Leggatt, David R Minkley, Ruth E Withler, Terry D Beacham, Ben F Koop, Robert H Devlin

**Affiliations:** Fisheries and Oceans Canada, 4160 Marine Drive, West Vancouver, BC V7V 1N6, Canada; Department of Biology, University of Victoria, 3800 Finnerty Road, Victoria, BC V8W 2Y2, Canada; Fisheries and Oceans Canada, Pacific Biological Station, 3190 Hammond Bay Road, Nanaimo, BC V9T 6N7, Canada; Fisheries and Oceans Canada, 4160 Marine Drive, West Vancouver, BC V7V 1N6, Canada; Department of Biology, University of Victoria, 3800 Finnerty Road, Victoria, BC V8W 2Y2, Canada; Department of Biology, University of Victoria, 3800 Finnerty Road, Victoria, BC V8W 2Y2, Canada; Fisheries and Oceans Canada, 4160 Marine Drive, West Vancouver, BC V7V 1N6, Canada; Fisheries and Oceans Canada, 4160 Marine Drive, West Vancouver, BC V7V 1N6, Canada; Fisheries and Oceans Canada, Pacific Biological Station, 3190 Hammond Bay Road, Nanaimo, BC V9T 6N7, Canada; Department of Biology, University of Victoria, 3800 Finnerty Road, Victoria, BC V8W 2Y2, Canada; Fisheries and Oceans Canada, 4160 Marine Drive, West Vancouver, BC V7V 1N6, Canada; Department of Biology, University of Victoria, 3800 Finnerty Road, Victoria, BC V8W 2Y2, Canada; Fisheries and Oceans Canada, Pacific Biological Station, 3190 Hammond Bay Road, Nanaimo, BC V9T 6N7, Canada; Fisheries and Oceans Canada, Pacific Biological Station, 3190 Hammond Bay Road, Nanaimo, BC V9T 6N7, Canada; Department of Biology, University of Victoria, 3800 Finnerty Road, Victoria, BC V8W 2Y2, Canada; Fisheries and Oceans Canada, 4160 Marine Drive, West Vancouver, BC V7V 1N6, Canada

**Keywords:** genome duplication, genome assembly, whole genome re-sequencing, SNPs

## Abstract

Chum salmon are ecologically important to Pacific Ocean ecosystems and commercially important to fisheries. To improve the genetic resources available for this species, we sequenced and assembled the genome of a male chum salmon using Oxford Nanopore read technology and the Flye genome assembly software (contig N50: ∼2 Mbp, complete BUSCOs: ∼98.1%). We also resequenced the genomes of 59 chum salmon from hatchery sources to better characterize the genome assembly and the diversity of nucleotide variants impacting phenotype variation. With genomic sequences from a doubled haploid individual, we were able to identify regions of the genome assembly that have been collapsed due to high sequence similarity between homeologous (duplicated) chromosomes. The homeologous chromosomes are relics of an ancient salmonid-specific genome duplication. These regions were enriched with genes whose functions are related to the immune system and responses to toxins. From analyzing nucleotide variant annotations of the resequenced genomes, we were also able to identify genes that have increased levels of variants thought to moderately impact gene function. Genes related to the immune system and the detection of chemical stimuli (olfaction) had increased levels of these variants based on a gene ontology enrichment analysis. The tandem organization of many of the enriched genes raises the question of why they have this organization.

## Introduction

Pacific salmon are keystone species with native habitat encompassing the northern Pacific Ocean and surrounding drainages (Willson and Halupka 1995). Most ecotypes of the various salmon species migrate to the ocean to reach a mature size before their migration back to freshwater to spawn (Beamish 2018). When salmon do migrate back to natal streams or lakes, they provide a valuable source of food to many marine and terrestrial predators and scavengers (Helfield and Naiman 2006). Some of these salmon spawning and migratory events are so large that they can alter the chemistry of streams for months at a time and even whole ecosystems (Helfield and Naiman 2001, 2006; Chaloner and Wipfli 2002; Hood *et al*. 2007; Janetski *et al*. 2009; Field and Reynolds 2011; Toge *et al*. 2011).

Chum salmon are the second largest of the Pacific salmon and may have historically represented up to 50% of the salmonid biomass in the Pacific Ocean (Salo 1991). With a habitat ranging from streams in Japan, the eastern coast of the Korean Peninsula, northern Russia, and all the way from the Mackenzie River in Canada to central California in the United States, chum salmon have the widest natural distribution of the Pacific salmon (Behnke and McGuane 2002). They have also been found to be the most plentiful species by weight in 3 out of 4 years between 2012 and 2015 and the second most valuable by processed value when averaged in this time period from an analysis of British Columbia fisheries (Gislason *et al*. 2017).

Chum salmon genetic resources have, as in many other species, been in a state of transition as genetic tools have advanced and become more widespread. Early work on population genetic structure in chum salmon utilized allozymes (Phelps *et al*. 1994; Seeb and Crane 1999) and microsatellite markers (Beacham *et al*. 2008) and provided the first range-wide studies on genetic diversity (Beacham *et al*. 2009). More recently, genetic stock identification tools have been shifting from microsatellites to single nucleotide polymorphisms (SNPs), providing increased accuracy of genetic discrimination with increasing marker numbers (Smith and Seeb 2008). Early identification of SNPs in chum salmon (Smith, Baker, *et al*. 2005; Smith, Elfstrom, *et al*. 2005; Elfstrom *et al*. 2007; Seeb *et al*. 2011; Petrou *et al*. 2013) led to the development of an SNP panel for assessing genetic diversity and population structures in chum salmon (Small *et al*. 2015). Development of expanded SNP panels for fisheries management continues to occur with increased marker density and improving genetic resources (Terry D. Beacham, Fisheries and Oceans Canada—personal communication).

Much of what we understand of the chum salmon genome, besides some basic characteristics and the structure of its 37 chromosomes (Phillips and Ráb 2001; Phillips *et al*. 2007; Waples *et al*. 2016), comes from its complicated and fascinating history that is shared with other salmonid species. Chum salmon share an ancestral whole-genome duplication with all other salmonids. The genome duplication occurred in a common ancestor of salmon around 90 million years ago and was a duplication of the existing ancestral genome (autopolyploidy) rather than a copy originating from another closely related species (allopolyploidy; Allendorf and Thorgaard 1984; Berthelot *et al*. 2014; Macqueen and Johnston 2014). Large portions of the duplicated genome have since diverged to a point that they might be considered as having residual tetraploidy (more similar to allopolyploidy) rather than being autotetraploid. In terms of percent sequence identity, most homeologous regions of the various salmon genomes have roughly 85–95% identity with each other (Lien *et al*. 2016; Christensen *et al*. 2018, 2020, 2021). However, there are particular homeologous chromosomes that still have cross-over events during meiosis, which allows exchange between homeologous chromosomes (Allendorf and Thorgaard 1984; Allendorf *et al*. 2015). These regions may have much higher sequence identity (Lien *et al*. 2016; Christensen *et al*. 2018, 2020, 2021), but exactly how similar or to what extent is still not well understood as sequencing technology has been a limiting factor. High error rates, restricted sequence lengths, and computation methods not optimized for residual autotetraploidy have all limited our ability to fully resolve these regions in the past. However, advances in this area continue with some modern salmonid genome assemblies now having nearly fully contiguous chromosome sequences (e.g. rainbow trout, *O. mykiss,* GCF_013265735.2 (Gao *et al*. 2021), and Atlantic salmon, *Salmo salar*, GCF_905237065.1).

Whole-genome duplications (WGDs) are significant evolutionary events as the extra copy of the genome can have reduced selective constraints allowing copies to rapidly evolve (Ohno 1970; Otto and Whitton 2000; Taylor and Raes 2004; Crow and Wagner 2006). Genome duplications have been proposed to allow for adaptations to new niches or conditions, particularly in times of major environmental change (reviewed in Van de Peer *et al*. 2017). The occurrence of over 70 different salmonid species lineages stemming from the ancestral WGD (Macqueen and Johnston 2014) offers a valuable system to (1) observe evolutionary consequences of an autopolyploid WGD, (2) identify ensuing mechanisms for regaining stable meiosis and cell division by regaining a functional diploid state through re-diploidization, (3) draw associations between mechanisms of re-diploidization to the potential genetic specialization that allows for species adaptation such as disease resistance, and (4) explore if residual tetraploid regions with high sequence similarity have an enrichment of particular genes that might be driving the retention of these highly similar homeologous regions. We focus on the last topic in the present study to better understand the influence of a WGD on the biology of chum salmon.

A fully-annotated reference chum salmon genome assembly will also enhance the development of genomics-based technologies to improve the effectiveness of fisheries management of the chum salmon fishery (e.g. through improved SNP panels that can target specific genes, genomic loci, or ecologically important markers identified in other species). SNP panels with improved performance at distinguishing populations, necessary for effective management of a fishery, have already been created for other Pacific salmon species in British Columbia (e.g. Beacham *et al*. 2020, 2021), and a genome assembly for chum salmon would provide the ability to adopt similar management tools based on emerging high-throughput sequencing technologies. SNP panel development benefits from the use of a reference genome assembly as markers can be filtered to avoid repetitive elements, and the genomic context is known—giving the ability to target or avoid genomic regions and avoid clustering markers. Transferring knowledge between projects is also easier with reference genome assemblies as a standard position and name are known for genetic loci.

To help achieve these goals, we sequenced the DNA from a male chum salmon from the Puntledge River Hatchery (the Puntledge River location can be viewed in Supplementary Fig. 1) using Nanopore sequencing to produce a contiguous and polished genome assembly (Illumina data from another project was also used for polishing). We were then able to map contigs/scaffolds to pseudo-chromosomes using a previously generated genetic map (family from Hoodsport Hatchery in Washington State, USA, Waples *et al*. 2016), and using synteny from the highly contiguous rainbow trout genome assembly (Gao *et al*. 2021). Whole-genome resequencing was performed on 59 individual hatchery chum salmon from a select distribution of the native range of this species to catalog genome-wide diversity, provide a common resource for other researchers to expand upon, and to better understand characteristics of the genome assembly.

## Materials and methods

### Animal care

Where rearing was required, animals were reared at Fisheries and Oceans Canada's Pacific Science Enterprise Centre in compliance with the Canadian Council on Animal Care Guidelines, under oversight from the Fisheries and Oceans Canada Pacific Region Animal Care Committee (for government personnel).

### Genome assembly

A spawning male Puntledge River Hatchery chum salmon (British Columbia, Canada: NCBI BioSample SAMN16447169, BioProject PRJNA669401) was collected and euthanized by Puntledge River Hatchery staff on 2019 October 30. Several tissues were collected and frozen on dry ice. The tissues were stored at −70°C.

A Nanobind Tissue kit (PacBio, formerly Circulomics) was used to extract high-molecular-weight DNA from the frozen tissues following the manufacturer's protocol with needle sheering. An SRE-XS kit (PacBio) was used to reduce small fragment of DNA from the extraction. Sequencing libraries were generated from the DNA extractions with the SQK-LSK109 kit (Oxford Nanopore Technologies—ONT) following the manufacturer's directions. FLO-MIN106D flowcells (ONT) were used to sequence the libraries on a MinION (ONT). FASTQ files were generated with the Guppy Basecalling Software (version 3.4.3 ONT) with default settings. Raw sequences were deposited to the NCBI SRA database under the SRX9304573 accession.

Flye (version 2.7b; Kolmogorov *et al*. 2019) was used to assemble the combined raw Nanopore sequences with default settings except that the expected genome size was set to 2.4 Gbp (read N50: ∼20 kbp, estimated read coverage: ∼29×). The assembly was then polished using Racon (version 1.4.16; Vaser *et al*. 2017) with default settings (alignments made with minimap2 version 2.13, Li 2018). The assembly was then polished two more times with trimmed Illumina paired-end reads using the default settings of the Pilon program (Walker *et al*. 2014). A similar method was used to produce the pink salmon (*O. gorbuscha*) genome assembly (Christensen *et al*. 2021), except Hi-C data was unavailable for scaffolding for the chum salmon (this means there will be an increase of order and orientation errors in the current genome assembly and could possibly alter the scaffold N50 when compared to the pink salmon). The Illumina paired-end data (Illumina HiSeq 2500) came from a Chehalis River Hatchery (British Columbia, Canada) chum salmon as it was already available (NCBI SRA accessions: SRX6595850-SRX6595851). The Illumina sequences were trimmed using trimmomatic (version 0.38; Bolger *et al*. 2014) with the following parameters: leading:28, trailing:28, slidingwindow:4:15, minlen:235, and the TruSeq3-PE adapters were included. The trimmed reads were then aligned to the genome assembly with bwa mem (version 0.7.17; Li and Durbin 2009; Li 2013 with the -M parameter and sorted and indexed with SAMtools version 1.9; Li *et al*. 2009).

Contig and scaffold placement onto pseudo-molecules was performed using Chromonomer (version 1.10; Catchen *et al*. 2020) with the disable_splitting parameter and a previously published genetic map (Waples *et al*. 2016). Ragtag (version 1.0.1; Alonge *et al*. 2019, 2022) was also used to generate pseudo-molecules based on synteny to the rainbow trout genome assembly (NCBI: GCF_013265735.2, Gao *et al*. 2021), which was more complete than the other salmonid genome assemblies at the time this protocol was performed. The contig/scaffold placements of both programs were checked manually, and preference was given based on the order of the genetic map. A final check for the placement of contigs/scaffolds was performed with alignments to the previous and unpublished chum salmon genome assembly (GCF_012931545.1) using the CHROMEISTER software (Pérez-Wohlfeil *et al*. 2019) with default settings.

### Whole-genome resequencing

Most samples were taken through the non-lethal collection of fin clips or operculum punches from Fisheries and Oceans Canada hatchery brood programs (by hatchery personnel). Additional samples were obtained from archived tissue sets used for genetic stock ID (Fisheries and Oceans Canada). All samples were from hatchery sources. While hatchery stocks may not accurately reflect natural populations in terms of diversity metrics and other population-level metrics, we were more focused on characterizing the genome and understanding which genes show increased diversity. Modern hatchery programs tend to take brood from nearby sources (Heard 2012), and our results should reflect global diversity if not local diversity. One of these samples (from Inch Creek Hatchery) was a doubled haploid (with no allelic variation) and was generated using the method from Biagi *et al*. (2022). In total, 59 individuals were utilized in this assessment (Supplementary File 1), with DNA obtained using the Qiagen DNeasy Animal tissue kit (following the manufacturer's protocol), or with a phenol/chloroform extraction (Thermofisher.com).

**Table 1.** Genome assembly metrics.

Genome size	2.6 Gb
Contig N50	2 Mbp
Number of contigs	17,497
BUSCO	98.1% complete 59.8% single-copy 38.3% duplicated 0.8% fragmented 1.1% missing
Protein-coding gene annotations	40,661
Masked with WindowMasker	53.86%

Values as reported by the NCBI.

**Table 2.** Summary of all sample SNP impacts and functional classes defined by snpEff.

Impact classification (counts of annotations)		Functional classification (counts of annotations)	
High impact	5,301 (853 genes)	Nonsense	2,177
Moderate impact	241,142 (22,914 genes)	Missense	242,014
Low impact	393,893 (31,806 genes)	Silent	302,152

Parentheses are the number of genes impacted.

Whole-genome resequencing libraries were prepared at the McGill University and Génome Québec Innovation Centre. Libraries were prepared using a NxSeq AmpFREE low DNA Library Kit and NxSeq Adapters (Lucigen). The libraries were then sequenced on an Illumina HiSeq X (PE150). Raw reads were uploaded to the NCBI (BioProject PRJNA556729).

### SNP calling, filtering, and annotation

BWA mem (version 0.7.17, parameters: -M, Li 2013) was used to align reads from each resequenced genome to the genome assembly (GCF_023373465.1), and SAMtools (version 1.12, parameters: default, Li *et al*. 2009) was used to sort and index the resulting alignment file. Picard (version 2.26.3, parameters: default, “Picard” 2020) was used to add read-group information and then mark PCR duplicates. Individual gVCFs were produced using GATK (version 3.8, parameters: genotyping mode discovery and emitRefConfidence GVCF, McKenna *et al*. 2010; DePristo *et al*. 2011; Van der Auwera *et al*. 2013), and a combined VCF was also produced using GATK (parameters: max alternate alleles 3). As a truth SNP dataset was unavailable for chum salmon, we did not recalibrate SNP scores.

Instead, we used several filters to reduce the number of false-positive variants. First, indels, loci with > 10% missing genotypes, and SNPs with multiple alleles were filtered using VCFtools (Danecek *et al*. 2011). To further reduce the number of false-positive variant calls, we filtered variants that were heterozygous in the doubled haploid individual, as these were likely from variants between repetitive elements or other collapsed regions of the genome where a proportion of reads would map erroneously. To filter for sequencing errors, a minor allele frequency of 0.05 was used and each locus needed an average coverage between 5 and 100. These filters were applied using VCFtools. For a principal component analysis, we also filtered for linkage disequilibrium using a BCFtools (version 1.9; Danecek *et al*. 2021) plugin (parameters: +prune, -w 20 kb, -l 0.4, -n 2). The R (version 4.2.1; R Core Team 2022) programs adegenet (version 2.1.7; Jombart 2008; Jombart and Ahmed 2011), ggplot2 (version 3.3.6; Wickham 2016), and vcfR (version 1.13.0; Knaus and Grünwald 2017) were used to perform the principal component analysis and plot the results.

SnpEff (version 5.0e; Cingolani *et al*. 2012) was used to generate an annotation database from the NCBI's GCF_023373465.1 GTF and sequence files. The filtered SNPs were then annotated using the same program. We identified genes with different SNP impact annotations using a Python script (Christensen 2023b). For each individual, we also generated a subset of heterozygous loci using VCFtools and annotated them to obtain metrics for each individual.

### Circos plot

Homeologous regions between chromosomes and the similarity between the two regions were identified with a pipeline utilizing BLAST (version 2.12.0+; Camacho *et al*. 2009; Chen *et al*. 2015) and Python scripts (Christensen 2022). A bar graph of repetitive elements along each chromosome was generated from the NCBI repeat-masked version of the genome assembly with a Python script (Christensen 2021). A chum salmon genetic map that was used in the genome assembly (Waples *et al*. 2016) was mapped to the genome using BLAST. Markers that had a minimum percent identity of less than 80% were filtered, and only the best location was kept. These data were plotted with Circos software (Krzywinski *et al*. 2009).

Eight regions with high homeologous sequence identity were identified by adding the total alignment lengths for each region together (Supplementary File 2). There were eight homeologous regions that had alignment lengths greater than 15 Mbp. All other regions had lengths less than 10 Mbp. We note, however, that the homeologous region between linkage group (LG) 10 and LG 32 had a similar density of alignments as the eight regions with lengths greater than 15 Mbp.

Collapsed regions of the genome assembly were identified by analyzing the alignment file of a doubled haploid individual using a Python script (Christensen 2023a). This script averaged read alignment coverage for each 1 Mbp window in the assembly. Collapsed regions were defined as those that had an average coverage of two standard deviations from the average of all other regions. This script was also used to analyze 10 kbp windows identified in the genome-wide association (GWA) analysis below.

### Gene ontology enrichment

Gene ontology (GO) terms were mapped to the NCBI annotated protein-coding genes with a pipeline using BLAST, the reviewed UniProtKB Swiss-Prot database (downloaded 2023 January 19; The UniProt Consortium 2023), the ID mapping service from UniProt, and python scripts (pipeline available at Christensen 2023c). Gene subsets were then analyzed for enriched GO categories using Ontologizer (version 2.1; Grossmann *et al*. 2007). All genes with assigned GO terms were used as the background set and the Benjamini-Hochberg (FDR) method was used for multiple test correction (*P*-value ≤ 0.05 after correction was chosen as the threshold for enrichment).

Enrichment tests were performed for gene subsets in collapsed homeologous regions greater than 3 Mbp, a subset of genes with high impact SNP annotations, a gene subset with moderate impact annotations with a density of 1.8557 variants/kbp or greater (2 standard deviations from the average of 0.34), and with a subset of genes with 13 or more total moderate impact annotations (2 standard deviations from the average of 3.2). The moderate impact annotation density was calculated as the number of moderate SNP annotations divided by the total gene length. Gene lengths were taken from the NCBI annotation file. For enriched GO terms in the collapsed homeologous regions, REVIGO (Supek *et al*. 2011) was also used to reduce the number of enriched GO terms reported in the main figure (using the small option and default settings).

### Sex determination

The *sdY* gene is the sex-determining gene in many salmonids (Yano *et al*. 2012, 2013; Bertho *et al*. 2018). We used BLAST to localize this gene to the NW_026282589.1 unplaced contig (aligning the Chinook salmon, *O. tshawytscha*, *sdY* NCBI accession: KC756279.2 to the genome assembly). From inspections of the unfiltered VCF file using IGV (Thorvaldsdóttir *et al*. 2013), we observed that females had missing genotypes in this region and that males had homozygous/hemizygous genotypes except in a region with a repetitive element (determined from the alignment file of the female doubled haploid individual).

Since this contig was unplaced, we used a GWA analysis with sex as the phenotype to determine the LG where this scaffold belonged, i.e. the X or Y-chromosome. We used logistic regression in PLINK (version 1.9; Chang *et al*. 2015) to perform the analysis (parameters: logistic mperm = 1000, covar to add sampling site as a covariate, ɑ = 0.05 after correction), and plotted the results using R and the package qqman (version 0.1.8; Turner 2014, 2018). Logistic regression was used as the trait was binary in nature and covariates needed to be added. A permutation test was used to correct for multiple testing, and the sampling site was added as a covariate to control for unknown population structure. We also performed clustering based on missing genotypes in PLINK (parameter: –cluster missing) to identify hidden structure in the data that might influence the GWA. All individuals clustered into the same group suggesting that missing data would not greatly influence the analysis.

There were three peaks identified in the GWA analysis, but we suspected that two of these might be false positives. We examined the read coverage of these regions (Christensen 2023a) of a male and female sample to determine if repetitive elements from the Y-chromosome were mapping erroneously to autosomal regions (in which case we expect to observe an increased coverage in males but not in females because females do not have the Y-chromosome). Repetitive elements can be collapsed during genome assembly and mis-mapping during read alignment can generate false-positive associations, where the variation is between repetitive element paralogous sequence variants rather than between alleles. If nucleotide variants are unique to repetitive elements on the Y-chromosome, mis-mapped reads on the autosomal repetitive elements will incorrectly generate associations with sex. We also aligned example flanking sequences from SNPs associated with sex (with the lowest *P*-value) from the suspected false-positive regions to the chum salmon RefSeq Representative genome assembly using blastn (default settings). If there were more than two alignments with exact matches, the SNPs were considered to be in repetitive elements.

## Results

### Genome assembly

**Fig. 1.**
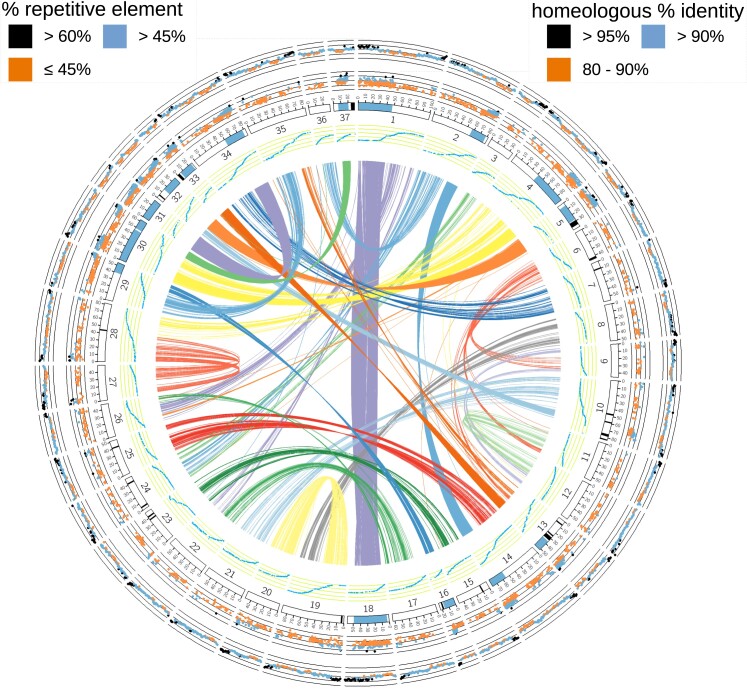
Circos plot of the chum salmon genome assembly. The links in the middle show alignments longer than 10 kb between homeologous regions. There is a dotplot/Marey map of genetic markers from the genetic map (Waples *et al*. 2016) used to place scaffolds in the next ring. The *X*-axis represents the nucleotide positions and the *Y*-axis represents the centimorgan position of a linkage group. The next ring displays the linkage groups/chromosomes. The blue highlights represent highly similar regions between homeologous chromosomes and the black regions (darker shade) of the genome assembly that were collapsed. On the next ring is the percent identity of the alignments between homeologous chromosomes (range: 80-100%). Points that are orange represent percent identities >80 and ≤90% (bottom half), blue >90 and ≤95%, and black >95% (top quarter). Repetitive elements are represented on the outermost ring. Windows (1 Mbp) with ≤45% repeat DNA are orange, blue >45% and ≤60%, and black if >60% (darker shade).

The chum salmon genome assembly (NCBI accession: GCF_023373465.1) is 2.6 Gb in length, contains 40,661 protein-coding gene annotations, and has 98.1% complete BUSCOs (59.8% single-copy, 38.3% duplicated; Table 1). In total, there were 66,713 gene annotations with approximately 61% protein-coding, 34% that were non-coding, 5% pseudogenes, and a small compliment of other annotations (NCBI annotation report: GCF_023373465.1-RS_2023_03). Much of the chum salmon genome remains duplicated from the ancestral whole-genome duplication common to all salmonids, which can be observed by the number of alignments between homeologs (Fig. 1). This can also be observed by the high percentage of duplicated BUSCOs (Table 1). Individual alignments between homeologous regions ranged in length from ∼10 to ∼442 kbp (∼60 kbp average). The percent identity ranged between 82.29 to 99.56% (average of 90.93%).

**Fig. 2.**
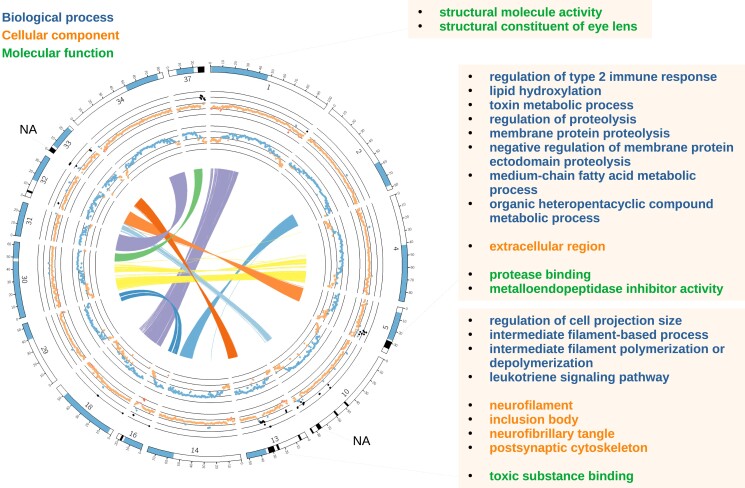
Circos plot of collapsed regions of the genome assembly. All linkage groups from Fig. 1 with highly similar homeologous sequences are shown. Homeologous alignments longer than 10 kbp are shown in the middle. The next ring out from the middle depicts alignment scores of reads from the resequenced genome of a doubled haploid female chum salmon (Inch Creek Hatchery stock) in windows of 1 Mbp. Average scores >20 and ≤40 were plotted as orange (bottom half), and values >40 and ≤60 were blue. The next ring shows the coverage of the same chum salmon reads in 1 Mbp windows. The color of each point is based on the standard deviation from the average coverage over all points. The red color represents >1 standard deviation and <2 standard deviations below the average (lower darker shade). The orange color represents ≤1 standard deviation below the average and <1 standard deviation above the average. Blue represents <2 standard deviations above the average (upper darker shade) and black represents ≥2 standard deviations above the average (upper darkest shade). On the outer ring, the linkage groups are highlighted with blue for regions that are highly homeologous and black (darker shade) where they are collapsed in the genome assembly. Gene enrichment was tested for collapsed regions ≥3 Mbp. If enriched GO terms were identified, they are shown (biological process—blue or top, cellular component—orange or middle, molecular function—green or bottom. Note: LG37 only contains GO terms for molecular function).

Eight homeologous region pairs have remained highly similar. This can be observed from the density of alignments between them (Fig. 1), and because they all have combined alignment lengths of more than 15 Mbp. From an analysis of the coverage of mapped reads from a doubled haploid chum salmon, we observed that four of the eight major homeologous regions of the chum salmon genome assembly had each collapsed for at least 3 Mbp into one sequence during assembly (Fig. 2). This is possibly a result of the high error rate of the sequencing technology combined with the high percent identity of these regions (Fig. 1). Together, all of the collapsed regions represent around 51 Mbp or ∼2% of the genome assembly length. This is likely an underestimate as unplaced contigs/scaffolds were not analyzed. Unplaced contigs/scaffolds may be enriched for highly similar homeologous sequences since the assembly program would have difficulty separating them. Placing these scaffolds would also suffer with more than one mapping location possible.

### GO in collapsed homeologous chromosomes

We investigated if there were GO categories enriched in the five major collapsed regions of the genome assembly (Fig. 2). There were 349 genes with GO term annotations (out of 33,448 genes with GO term annotations genome-wide) in the collapsed homeologous regions larger than 3 Mbp (Fig. 2, Supplementary File 2). The enriched GO terms with the lowest adjusted *P*-values from these regions were *negative regulation of membrane protein ectodomain proteolysis* (*P*-value = 6.02E−07, 6 study terms out of 86 in the genome, LG 5), *intermediate filament polymerization or depolymerization* (*P*-value = 2.93E-04, 3 study terms out of 4 in the genome, LG 13), and *structural constituent of eye lens* (*P*-value = 4.84E-05, 10 study terms out of 82 in the genome, LG 37). Other enriched categories are noted in Fig. 2 (reduced using REVIGO) and the full set is in Supplementary File 2.

### SNP annotations and GO

**Fig. 3.**
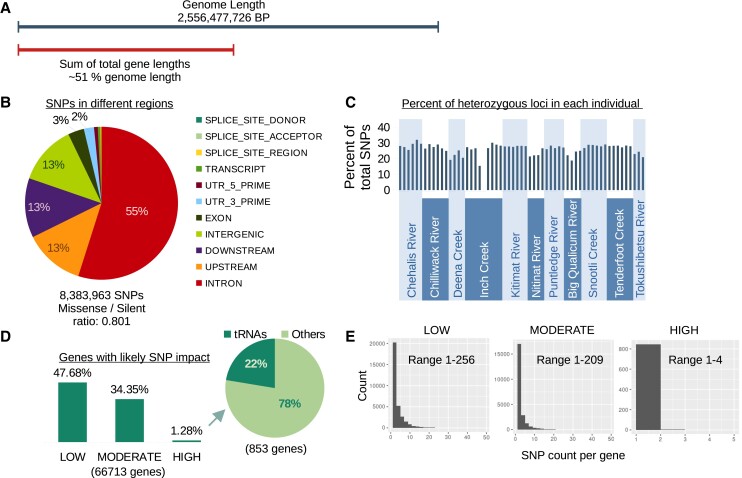
Characteristics of SNPs identified from resequenced genomes. a) The genome length compared to the combined length of all of the annotated genes (including the lengths of introns). b) A pie chart of the snpEff annotations. SNPs can have multiple annotations. c) A barplot of the percent of heterozygous loci per individual. The bars with an underline are from female salmon. d) Left: A barplot of the percent of genes with at least one variant that has a predicted low, moderate, or high impact on gene function. Right: A pie chart of the genes with at least one high-impact SNP. e) Histograms of the counts of low, moderate, and high variants in each gene. Most genes only have one of these variants.

To better understand the expanse of nucleotide diversity in the sampled chum salmon, SNPs (8,383,963 after filtering) were annotated for genomic location (e.g. intron) and potential impact on gene function (e.g. missense) (Table 2). Around 55% of the 28,683,938 SNP annotations from the resequenced genomes were located in introns (Fig. 3). SNPs may have multiple annotations as they can influence more than one gene or transcript variant. For comparison of the 55% of annotations in introns, the sum of gene lengths was ∼51% of the genome length (Fig. 3). The mean length of annotated genes was 21,934 bp. Another ∼38% of the annotations were located in intergenic regions (downstream, upstream, and intergenic categories), and only ∼3% were in exons (Fig. 3). The percent of heterozygous variants per individual ranged from zero (in the doubled haploid individual) to ∼32% (Fig. 3). A PCA analysis of the individuals based on an SNP set filtered for linkage disequilibrium can be viewed in Supplementary Fig. 2.

To investigate the potential influence of the SNP variation in the sampled chum salmon, we identified genes with SNPs categorized as low, moderate, and high impact. These categories in snpEff predict if a variant might influence the function of a gene, e.g. a stop gained annotation would be given a high impact annotation. Around 48% of all genes had at least one SNP that was annotated as low impact in the sampled chum salmon (Fig. 3). There were ∼34% with a moderate impact, and only ∼1% of all genes were predicted to have an SNP with high impact (Fig. 3). Many of the genes with predicted high impact SNPs were tRNAs (Fig. 3).

There were between 1 and 256 low-impact SNP annotations per gene—for genes with these variants (Fig. 3). This range was 1–209 for moderate and 1–4 for high-impact annotations. There were no enriched GO terms of the 853 genes with at least one high-impact SNP. Only 261 of the 853 genes had GO annotations before the enrichment analysis was performed. This may reflect the high number of non-coding genes without GO annotations in this group.

**Fig. 4.**
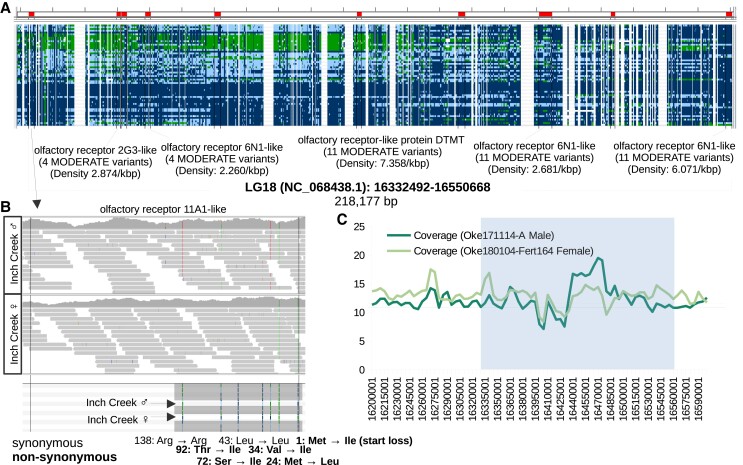
Genes with high-density moderate impact SNPs. a) Screenshot from IGV of tandem olfactory receptor genes with high-density SNPs with moderate predicted impacts on gene function. The red highlights on the top show the boundary of the various olfactory receptor genes. Each column represents a variant and each row is a resequenced genome (ordered to highlight haploblocks within the region). Dark blue genotypes (darkest shade) are homozygous for the reference allele, light blue for heterozygous genotypes (lightest shade), and green for homozygous for the alternative allele (white for missing). b) Screenshot from IGV of the first gene from a. Top: Read alignments from a diploid male. Differences from the reference sequences are shown in color (darker shades than reads). A summary of coverage is displayed at the top. Middle: Same as the top, but for a doubled haploid female. Bottom: Called variants based on all individuals. Genotypes from the individuals on the top and middle are highlighted (see a for which color represents the various genotypes). The impact on amino acid sequences is noted below for each variant (in reverse order as the gene orientation is also reversed). c) Average read coverage in 10 kbp windows of the individuals from b. The average of each individual is shown on the graph with a horizontal line. The region from a is highlighted.

A GO term enrichment analysis of genes with moderate impact SNPs at high densities (moderate impact SNPs/total gene length * 1 kbp) was also performed with 644 genes identified. The GO term with the lowest adjusted *P*-value (1.04E-24) from this analysis was the *detection of chemical stimulus* (146 significantly enriched GO terms in total were identified, Supplementary File 3). Twenty of the 34 genes within the *detection of chemical stimulus* GO category (208 genome-wide) were either odorant (*n* = 1) or olfactory receptors (*n* = 19). Ten of the olfactory receptors were localized to a ∼200 kbp region on LG 18 (Fig. 4). The category with the second lowest adjusted *P*-value (7.72E-16) was *immunoglobulin complex* (13 genes out of 33 genome-wide, Supplementary File 3).

There were 384 enriched GO terms (Supplementary File 3) identified from the set of 691 genes (501 with GO annotations) with at least 13 moderate impact annotations per gene (13 moderate SNPs per gene was two standard deviations from the average of three, Supplementary File 3). This represents ∼3% of the genes with at least one moderate impact annotation (out of 22,914), but ∼20% of all of the moderate impact annotations (out of 73,405). The GO term with the lowest adjusted *P*-value from this analysis was the *negative regulation of NIK/NF-kappaB signaling* term (*P*-value = 1.80E-15, Supplementary File 3). There were 29 genes in this subset out of a possible 141 genes in the genome with this or an interacting GO term (Supplementary File 3). All of the genes belonged to the NOD-like receptor family (*NLRC3 n* = 12, *NLRP3 n* = 4, *NLRP7 n* = 1, *NLRP12 n* = 12). This indicates that ∼21% of the genes related to the *negative regulation of NIK/NF-kappaB signaling* GO category have at least 13 SNPs per gene from the moderate impact category. The GO term with the second lowest *P*-value from this analysis was the *MHC class I biosynthetic process* (*P*-value = 1.68E-13, 16 genes out of 50 genome-wide). Genes in this category were a subset of the *negative regulation of NIK/NF-kappaB signaling* GO category with the exception of one gene (Supplementary File 3).

**Fig. 5.**
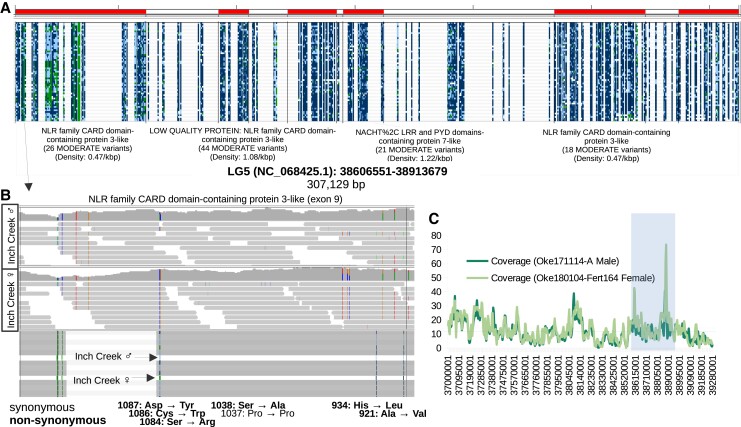
Genes with high counts of moderate impact SNPs. a) Screenshot from IGV of tandem NOD-like receptor family genes with high counts of moderate impact SNPs. The red highlights on the top show the boundary of the various genes. Each column represents a variant and each row a resequenced genome (ordered to highlight any haploblocks within the region). Dark blue genotypes (darkest shade) are homozygous for the reference allele, light blue for heterozygous genotypes (lightest shade), and green for homozygous for the alternative allele (white for missing). b) Screenshot from IGV of the first gene from a (exon 9). Top: Read alignments from a diploid male. Differences from the reference sequences are shown in color (darker shades than reads). A summary of coverage is displayed at the top. Middle: Same as the top, but for a doubled haploid female. Bottom: Called variants based on all individuals. Genotypes from the individuals on the top and middle are highlighted (see a for which color represents the various genotypes). The impact on amino acid sequences is noted below for each variant (in reverse order as the gene orientation is also reversed). c) Average read coverage in 10 kbp windows of the individuals from b. The average of each individual is shown on the graph with a horizontal line. The region from a is highlighted.

Two of the 29 genes with the assigned *negative regulation of NIK/NF-kappaB signaling* GO term (LOC127930349 and LOC118383248) were within a collapsed region of the genome assembly (LG 5). Six were nearby the same collapsed region (Fig. 5), and all but one were otherwise on unplaced scaffolds, which may be indicative of them belonging to a homeologous region that might have high sequence similarity with its duplicated region. There was evidence of mis-aligned reads (and likely unaligned reads) in this region as there was high variability in the read coverage, and heterozygous genotypes were identified in the mis-aligned reads from a doubled haploid chum salmon (Fig. 5). Even though this region was not identified as a collapsed region in the coverage analysis, this evidence suggests that at least some of this region is likely collapsed as well. The identified nucleotide variation in this region could be from both allelic variation and from variation between paralogs/homeologs. If we assume that all seven of the called SNPs were variants between paralogous sequences, that would be a percent identity of 98.74% between homeologous sequences. If we assume the called variants are alleles but the 5 heterozygous variants filtered during SNP calling and observed in the doubled haploid individual are paralogous, then the percent identity would be 99.10% (97.78% with all variants as paralogous sequence variants).

### Sex determination

It was previously discovered that *sdY* is the sex-determining gene in many salmonids (Yano *et al*. 2012). The data from this study is consistent with *sdY* being the sex-determining gene in the chum salmon sampled. All the females were missing genotypes on the contig with the *sdY* gene (NW_026282589.1) and all the males had homozygous/hemizygous genotypes (Supplementary Fig. 3). The *P*-value from a chi-square test of the sdY contig haplotypes (presence or absence) was 6.9144E−13, and no individuals deviated from expected genotypes based on their phenotype.

**Fig. 6.**
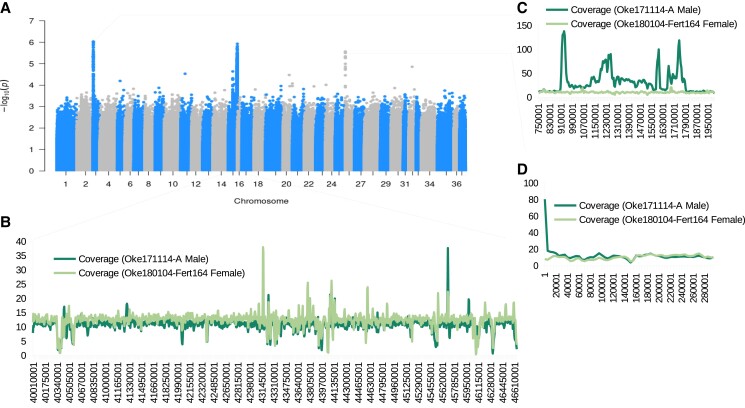
GWA analysis of sex. a) Manhattan plot of the GWA (only variants on chromosomes are shown). Each point represents an SNP on a chromosome, and the height represents the −log10 *P*-value of the SNPs association with sex (sampling site used as a covariate). All three peaks were significantly associated with sex (adjusted *P*-value ≤ 0.05, Supplementary Fig. 4). Each of the three peaks was evaluated for coverage. b) The average coverage (in 10 kbp windows) of a diploid male and a doubled haploid female in the peak on LG 15. c) Same as b for the peak on LG 3. d) Same as b for the peak on LG 26.

A GWA analysis was performed to find the most likely location of the *sdY* containing unplaced contig (NW_026282589.1). There were three peaks in the GWA analysis (Fig. 6, Supplementary Fig. 4). Two of the peaks were localized to relatively small distances (<200 kbp) compared to the third on LG 15 (>6 Mbp).

Coverage of the two narrower association peaks was uneven between the male and the female analyzed (Fig. 6). There was higher coverage in the male for these regions. This could be caused by repetitive elements unique to males aligning to these regions. In some windows, there was an average coverage of around 10 × higher in the male. Clustering based on missing data did not appear to be based on sex and should not influence this analysis (Supplementary Fig. 5).

We tested if the peaks on LG 3 and LG 26 could be caused by repetitive elements. We did this by aligning flanking sequences of SNPs, with the lowest *P*-values, to the reference genome assembly (Supplementary Fig. 6). The three SNPs tested were indeed within repetitive elements as they perfectly aligned to multiple locations within the genome assembly (Supplementary Fig. 6). This information points to LG 15 as the likely sex-chromosome and that the other signals are possibly false positives caused by mis-mapping, which is consistent with McKinney *et al*. (2020).

## Discussion

### Genome assembly

The chum salmon genome assembly is highly contiguous and contains the vast majority of the expected genic sequence as determined by a BUSCO analysis. These metrics suggest this assembly is suitable for many applications and is similar to the metrics of other salmon genome assemblies (e.g. Christensen *et al*. 2021; Rondeau *et al*. 2023). The number of annotated protein-coding genes in the Pacific salmon with available annotated genome assemblies, for example, ranged from 37,942 in sockeye salmon, *O. nerka* (GCF_006149115.2, Christensen *et al*. 2020) to 41,269 in the coho salmon, *O. kisutch* (GCF_002021735.2, Rondeau *et al*. 2023). The chum salmon assembly is intermediate with 40,661. The percent of protein-coding genes relative to the total number of genes ranged from 61% in chum salmon to 83% in sockeye salmon (coho salmon: 65%, pink salmon: 68%, and Chinook salmon: 79%). These values are unlikely to be reflective of differences in actual gene number among species but represent differences in genome assembly quality, underlying annotation data, or the annotation software version that differed among all genome assembly versions except between pink and Chinook salmon.

A portion (at least 2%) of the genome assembly was collapsed due to high sequence similarity between homeologous regions. This value probably underestimates the prevalence of collapsed regions as these regions are the most difficult to assemble and place on pseudo-chromosomes. One estimate of highly similar sequences in salmonid genomes suggests this value might actually be as high as ∼20% (Gidskehaug *et al*. 2011; Allendorf *et al*. 2015). While other research on salmonid genomes (e.g. Berthelot *et al*. 2014; Lien *et al*. 2016; Christensen *et al*. 2018, 2021; De-Kayne *et al*. 2020; Gao *et al*. 2021) has not quantified the proportion of collapsed homeologous regions as is performed here, we anticipate that even without the relatively high sequencing errors of long-read technology, assembly software would have difficulty disambiguating allelic variation from paralogous sequence variants in extremely similar homeologous sequence. These sections would likely be collapsed in at least a portion of the genome assembly (depending on the length of the sequences). Improved sequencing technologies, new assembly techniques, the use of doubled haploid samples, and increased resource allocation could all at least partially address this issue in future genome assembly versions. The most recent Atlantic salmon (GCF_905237065.1) and rainbow trout (Gao *et al*. 2021) assemblies are much more contiguous—possibly as a result of higher coverage and more accurate read technology.

The collapsed regions present an opportunity to better characterize highly similar homeologous regions and nucleotide variation in these regions. However, the start of collapsed regions and the percent identity when the assembly collapses is challenging to quantify. From the coverage analysis of a doubled haploid individual, for example, we observed that the collapsed regions are typically near transitions from a read coverage that is around the mean for the genome to a much higher coverage in the collapsed region itself. These transition regions may be where the assembly software starts to collapse some smaller sections and progressively increases the frequency and size of the collapsed segments. This is difficult to quantify as much of these sections may not actually be placed onto pseudo-chromosomes. Hi-C data may help to better quantify the actual percentage that has been collapsed.

In terms of percent identity, the genome assembly program was able to separate regions with as high as 99.56% sequence identity, but it remains unclear how consistently this was achieved and if chimeras were created. In exon 9 (554 bp) of the NLR family CARD domain-containing protein 3-like gene (LOC118380914), for example, there was evidence that this region was collapsed based on heterozygous genotypes in a doubled haploid individual (caused by mapping both homeologs to the collapsed sequence). The percent identity in this exon ranged from ∼98% to 99% between homeologous sequences depending on assumptions relating to which variants were allelic and paralogous sequence variants. This estimate was based on variation in the entire set of samples in this study. The percent identity might be higher on an individual basis.

There are at least eight highly similar homeologous regions of the salmonid genomes that have been described here and previously (e.g. Kodama *et al*. 2014; Sutherland *et al*. 2016). Four of the five largest collapsed regions were on these eight residually tetraploid chromosome pairs. The collapsed regions contain a proportional number of protein-coding genes (349 collapsed genes with GO annotations out of 33,448 in the genome, or ∼2% after accounting for both copies) as the proportion of the genome assembly that was collapsed (∼2%). It has previously been reported that recombination still occurs between homeologous chromosomes in these regions, explaining how high sequence similarity could be retained for as long as ∼90 million years (reviewed in Allendorf *et al*. 2015).

### GO in collapsed homeologous chromosomes

We identified several categories of genes that were enriched in the collapsed regions of the genome. In two of the three collapsed regions, we identified enriched GO terms related to the immune system (*leukotriene signaling pathway* and *regulation of type 2 immune response*) and toxins (*toxin metabolic process* and *toxic substance binding*). We discuss the possible benefits of genes from these categories to be enriched in highly similar collapsed homeologous regions in the following paragraphs.

Homeologous regions that have diverged to the genome-wide average of ∼91% identity or more may still have functional and duplicated genes, but each of the gene copies is expected to have a unique cellular role or function as their sequences have also diverged (reviewed in Ohno 1970; Zhang 2003; Glasauer and Neuhauss 2014). Exceptions may occur when extra copies of a gene are beneficial. In the collapsed regions, where homeologous recombination events are thought to maintain very high sequence similarity, homeologous genes are more likely to be effectively allelic in nature. There are potentially four copies of a gene in these collapsed regions (two alleles of a gene and two alleles of the duplicate gene), and consequently, there is the possibility that genes in these regions have increased nucleotide diversity or expression (i.e. dosage effects) that could affect their function(s).

Standing genetic variation in genes related to the immune system may allow salmon to appropriately respond to novel pathogens or parasites in the variety of environments they encounter during vast migrations (e.g. Dionne *et al*. 2009). The major histocompatibility loci are classical examples of this phenomenon. In vertebrates, the major histocompatibility loci are highly polymorphic and they are likely under balancing selection due to the constant assault of pathogens (reviewed in Hughes and Yeager 1998; Bernatchez and Landry 2003; Piertney and Oliver 2006). The link with increased nucleotide diversity and immune system performance is fairly well documented with the major histocompatibility loci, but how extra copies of a gene related to the immune system might be beneficial is unclear (in terms of increased expression). However, we note that salmon with additional genome duplications do have observable differences in their immune response to pathogens (reviewed in Benfey 2016).

In addition to pathogens, salmon encounter a wide variety of toxins (reviewed in Monosson 2012; Ross *et al*. 2013). Some of these are from recent human activities (e.g. persistent organic contaminants, O’Neill *et al*. 2015; pesticides, Harris *et al*. 2008; and heavy metals, Davis *et al*. 2001), but others are from environmental factors such as nest site substrate composition (Ross *et al*. 2013) and microorganisms (Craig *et al*. 1968; Brusle 1995). These contaminants can have a diverse impact on the biology of salmon. Heavy metals, for example, can alter olfaction (reviewed in Tierney *et al*. 2010); when coho salmon were exposed to low levels of dissolved copper, researchers observed that the exposed salmon did not respond normally to olfactory alarm cues or to a predator (McIntyre *et al*. 2012). The relationship between environmental toxins and nucleotide diversity has been studied in a few species. In killifish (*Fundulus heteroclitus*), high genetic diversity and gene duplications played key roles in the successful adaptation of killifish to highly polluted environments (reviewed in Whitehead *et al*. 2017). Similar effects may be acting via loci in homeologous regions of the salmonid genome.

### SNP annotations and GO

We identified 8,383,963 filtered SNPs from the 59 resequenced chum salmon genomes. The percent of polymorphic variants per individual was on average ∼26%, but some of this variation is likely paralogous sequence variants rather than allelic. On average, there were ∼3.4 snpEff annotations per SNP because each variant could influence more than one gene or transcript variant. The vast majority of the SNP annotations were in introns or intergenic regions (∼94%). The high percent of SNPs in introns was likely a reflection of the length of the genes, which when combined represent ∼51% of the genome. While this information is important for understanding base levels of genetic diversity (e.g. the percent of heterozygous SNPs per genome), these values are geographically context dependent. Additional individuals or sampling locations would likely increase the number of variants identified. It is also unclear how false positives (e.g. paralogous sequence variants) influence these values.

The number of genes with at least one SNP predicted to have a low to high impact on gene function ranged from ∼48% to ∼1% respectively. As with many population genetics calculations, there are several assumptions underlying these annotations (e.g. impact is the same among species), but they also provide a useful and comparable statistic among species or populations. Another benefit is that we can identify genes with high genetic diversity, and determine when the variants are more likely to impact gene function and phenotype.

We did not identify any enriched GO categories of genes with high-impact variants but noted that many of the genes with high-impact variants were non-coding tRNAs. There are 267 annotated tRNA genes for alanine alone in the current version of the genome assembly (https://www.ncbi.nlm.nih.gov/gene). If there are a similar number of tRNAs for the other amino acids, that would be roughly 5,000–6,000 tRNAs genome-wide or ∼7–9% of all annotated genes (66,713). This does not account for the possibility of collapsed regions of the assembly with tRNAs, but is still well below the ∼22% of genes with high-impact variants that were identified as tRNAs. Nucleotide variation in tRNAs has been identified in other species (Sprague *et al*. 1977; Goodenbour and Pan 2006; Parisien *et al*. 2013) and can influence important biological traits. One well-studied example is that of tRNA alanine variants in the silkworm (*Bombyx mori*), where one variant is expressed exclusively in the silk gland and is thought to increase the production of a major constituent of silk (Meza *et al*. 1977; Sprague *et al*. 1977; Ouyang *et al*. 2000).

Enriched GO categories based on the density of moderate impact SNPs included the *detection of chemical stimulus* and *immunoglobulin complex*. Many of the genes in the *detection of chemical stimulus* GO category were olfactory receptors. In Atlantic salmon, there are around 60–77 olfactory receptors (Johnstone *et al*. 2012; Liu *et al*. 2021a), and around 73–75 in chum salmon (Palstra *et al*. 2015; https://www.ncbi.nlm.nih.gov/gene). These genes are thought to be important for natal homing (Johnstone *et al*. 2011; Palstra *et al*. 2015), predator avoidance (McIntyre *et al*. 2012), and other traits. In humans, there are hundreds of olfactory receptors in our genomes, and genetic diversity in these genes is associated with changes in odor perception (Niimura and Nei 2003; Trimmer *et al*. 2019). While there are many fewer olfactory receptors in ray-finned fishes than in humans (Liu *et al*. 2021a), diversity in these genes is important in both humans and ray-finned fishes. In whole-genome studies of diversity in humans and a ray-finned fish, olfactory receptors were identified as candidates for balancing selection (Alonso *et al*. 2008; Liu *et al*. 2021b) similar to major histocompatibility loci. The diversity of alleles in this group of genes could impart a range in odor perception in chum salmon as it does in humans. Further studies on this topic may help to reveal why these genes, and by extrapolation possibly odor perceptions, are so diverse in chum salmon.

There is an inherent bias in examining moderate impact SNP density because the analysis will detect smaller genes with fewer moderate impact SNPs more often than in larger genes with exons that are spread out. For this reason, we also examined total counts of moderate-impact SNPs. The top enriched GO categories based on the total count were *negative regulation of NIK/NF-kappaB signaling* and *MHC class I biosynthetic process*. Most of the genes in these two categories overlapped (*NLRC3*, *NLRP3*, *NLRP7*, *NLRP12—*belonging to the NOD-like receptor family). These genes were in a region of the genome assembly that is either partially or completely collapsed. This could mean that the moderate impact SNPs discovered are actually variations between homeologs rather than between alleles. However, because these homeologs have such high sequence identity, they might be considered allelic rather than as different genes.

The *NLRC3*, *NLRP3*, *NLRP7*, and *NLRP12* genes are all involved either as part of an inflammasome or during the regulation of inflammasomes (reviewed in Tuladhar and Kanneganti 2020; Zheng *et al*. 2020; Carriere *et al*. 2021). Inflammasomes are initiated in response to various stimuli, such as bacteria or virus infection, and result in the inflammatory response due to the production of interleukin 1β and 18 from precursors (Zheng *et al*. 2020). Inflammasomes are tightly regulated as autoimmune diseases are a consequence otherwise (Zheng *et al*. 2020). In ray-finned fishes, the NOD-like receptor genes have many more members than in mammals (reviewed in Chang *et al*. 2021), which may allow alternative regulation or function not observed in mammals. The increase in diversity of genes likely means that gene expression from these families must also be tightly regulated to respond appropriately to environmental and physiological ques.

Interestingly, some of the gene groups identified by analyzing moderate impact SNPs were organized in tandem sections of the genome. Is there some unique feature of the genome in these regions that increases genetic diversity (e.g. recombination hot spots)? One insight we have from studies of chromosome structure within the cell is that genes that require allele-specific regulation of multiple genes, such as the olfactory receptors, are regulated together in various types of chromosome structures (reviewed in Bashkirova and Lomvardas 2019). Perhaps the nature of highly controlled regulation of many genes benefits from this type of tandem organization and genes that require this type of regulation naturally have greater diversity.

### Sex determination

With a GWA analysis, we were able to further support that the sex chromosome of chum salmon is LG 15. From a coverage analysis and an analysis of sequences of markers on the alternative association peaks, it appeared that the off-targets from the GWA analysis could have been the result of repetitive elements mapping to collapsed autosomal regions. Likely, the collapsed repetitive elements originated from a Y-chromosome source as they were associated with sex. Placement of the sex-determining region to LG 15 is consistent with a previous sex-chromosome assignment in chum salmon (McKinney *et al*. 2020). While LG 15 was associated with sex in the current and a previous study, sex determination can be complex and show variation among populations of the same species (e.g. Christensen *et al*. 2020).

LG 15 is homeologous to the sex-chromosomes of sockeye salmon, coho salmon, and whitefish (Phillips *et al*. 2005; Gagnaire *et al*. 2013; Kodama *et al*. 2014; Sutherland *et al*. 2017), chromosome 3.2 in chum salmon vs. 3.1 based on a naming scheme relative to northern pike, *Esox lucius* (Sutherland *et al*. 2016). Translocation of the sex-determining region has been observed in other salmonids (e.g. Eisbrenner *et al*. 2014; Lubieniecki *et al*. 2015), and it is thought to occur via jumping or transposition as a transposable cassette (reviewed in Bertho *et al*. 2021). The translocation of the sex-determining region between chromosomes might help to partially explain how genetic markers for sex in one salmon species might perform poorly in other species as previously reported (Muttray *et al*. 2017). Partial (for tandem repeats) or complete disruption between linked markers might occur due to translocation. Also, changes in recombination dynamics might be expected based on different chromosome positions, and these could influence events such as the deletion or duplication of genetic markers.

## Conclusions

In this study, we generated a chromosome-level genome assembly of the chum salmon that has metrics comparable to other modern salmon genome assemblies. From the analysis of resequenced genomes, we were also able to better characterize highly similar regions between homeologous chromosomes from the salmonid-specific genome duplication. We identified an enrichment of immune and toxin-related genes in these regions and also discovered that some genes have high levels of variants that are expected to moderately impact function in at least one of these regions. We observed that there was an enrichment of genes with immune and olfactory functions that have high levels of moderate impact SNPs in the sampled salmon. In addition to improving our basic knowledge of the chum salmon genome structure, the present research enhances our capability to understand the genetics of this species’ populations which, in turn, provides an improved ability to manage the species effectively in an era where natural and anthropogenic factors are prevalent.

## Supplementary Material

jkad127_Supplementary_Data

## Data Availability

Oxford Nanopore reads used to generate the genome assembly were deposited in the NCBI sequence read archive (SRA)—BioProject PRJNA669401. Illumina reads used to polish the genome assembly can be found in the SRA using the SRX6595850 and SRX6595851 accessions. Reads from the resequenced genomes were also deposited to the SRA under BioProject PRJNA556729. The filtered nucleotide variants (8,383,963 bi-allelic SNPs) were deposited to the GSA Figshare portal: https://doi.org/10.25387/g3.22693411. Python scripts used in the analyses can be downloaded from https://github.com/KrisChristensen. Supplemental material available at G3 online.
