# Supplementary material for: Insights from a chum salmon (Oncorhynchus keta) genome assembly regarding whole-genome duplication and nucleotide variation influencing gene function
Source: G3 (Bethesda). 2023 Jun 9;13(8):jkad127. doi: 10.1093/g3journal/jkad127 (PMC10411575; doi:10.1093/g3journal/jkad127)
Supplement: jkad127_Supplementary_Data [file jkad127_supplementary_data.zip › Figure_S6_G3-2023-404248.pdf]

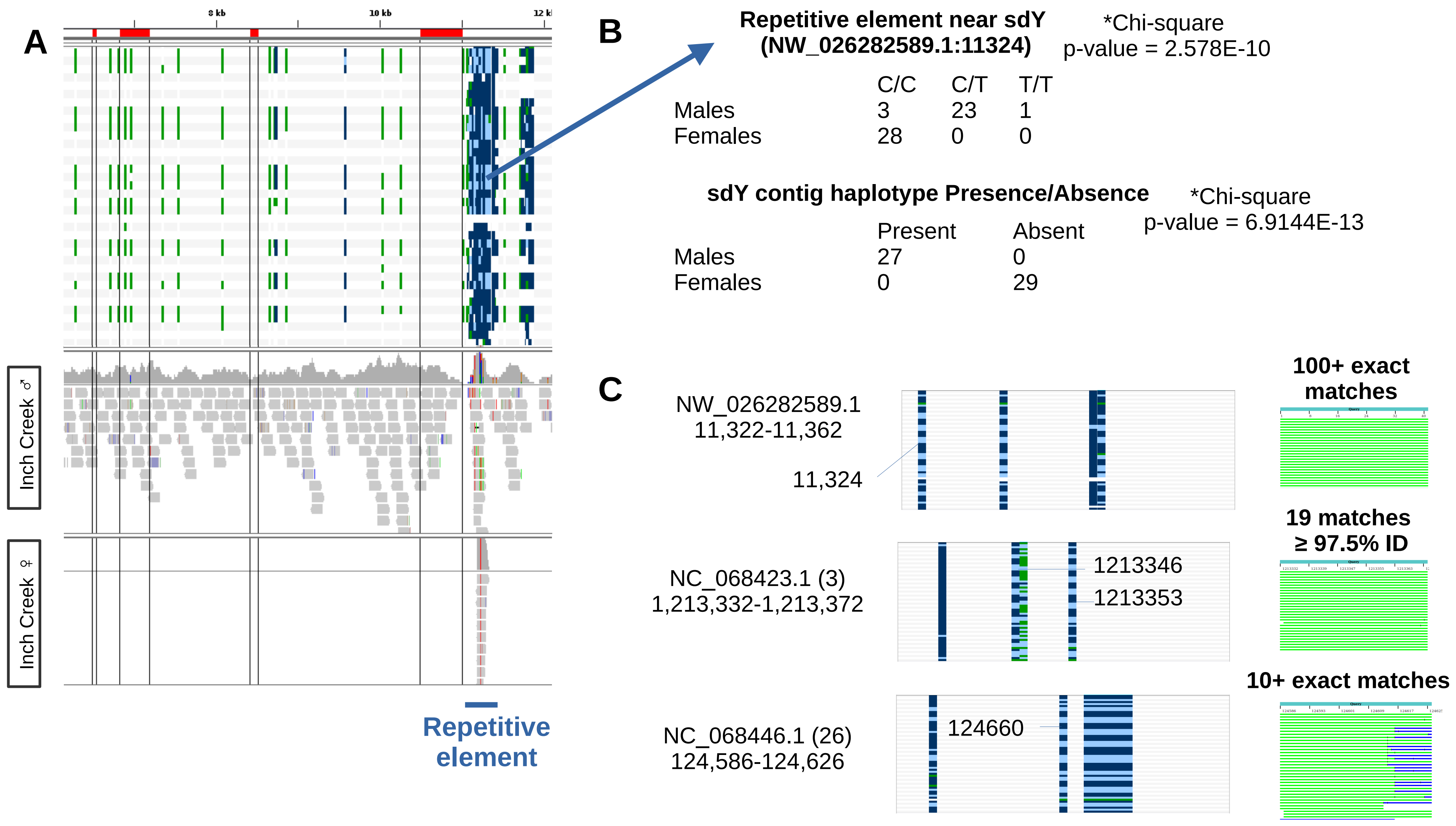
A
8 kb
10 kb
Inch Creek ♂
Inch Creek ♀
Repetitive element
B
Repetitive element near sdY
(NW_026282589.1:11324)
*Chi-square
p-value = 2.578E-10
C/C C/T T/T
Males 3 23 1
Females 28 0 0
sdY contig haplotype Presence/Absence
*Chi-square
p-value = 6.9144E-13
Present Absent
Males 27 0
Females 0 29
C
NW_026282589.1
11,322-11,362
11,324
100+ exact matches
NC_068423.1 (3)
1,213,332-1,213,372
1213346
1213353
19 matches ≥ 97.5% ID
NC_068446.1 (26)
124,586-124,626
124660
10+ exact matches
