# Supplementary material for: Insights from a chum salmon (Oncorhynchus keta) genome assembly regarding whole-genome duplication and nucleotide variation influencing gene function
Source: G3 (Bethesda). 2023 Jun 9;13(8):jkad127. doi: 10.1093/g3journal/jkad127 (PMC10411575; doi:10.1093/g3journal/jkad127)
Supplement: jkad127_Supplementary_Data [file jkad127_supplementary_data.zip › Supplemental_Material_Legends_G3-2023-404248.docx]

**Supplemental Material**

**Figure S1. Map of sampling locations.** All chum salmon are from hatchery sources. See File S1 for further details on samples.

**Figure S2. Principal component analysis of SNPs from chum salmon.** The SNPs used for this analysis were filtered for linkage disequilibrium.

**Figure S3. Contig with sdY gene.** A) Screenshot from IGV of contig NW_026282589.1 (NCBI accession). Each row represents an individual. Males are highlighted orange, but the sex of the sample can also be determined by the ending value of the sample name (far left), which is a letter for males. Each column represents a nucleotide variant. Missing genotypes are white, and females have missing genotypes for most of the length of this contig. The four exons of sdY (determined in B) were visualized by vertical lines and they have red highlights on the distance bar at the top. B) A screenshot of the pink salmon (which is the closest relative to chum salmon) sdY mRNA aligned to the sdY contig using Splign (Kapustin *et al.* 2008).

**Figure S4. Manhattan plot of sex genome-wide association analysis after corrections for multiple testing.** A) The *p*-values in this plot were corrected for multiple testing using 1000 permutations. The blue horizontal line represents an ɑ threshold of 0.05 and the red a 0.01 threshold. B) A qq-plot of unadjusted *p*-values from Figure 6. C) A qq-plot of the adjusted p-values (1000 permutations).

**Figure S5. Heatmap of identity-by-missingness matrix.** An identity-by-missingness matrix was generated in PLINK and then clustered using default settings of the pheatmap package (Kolde 2019). Sample identities are shown for each row and their corresponding sex is given for each column. The ratio between males and females is shown at the bottom for different clusters. In the PLINK analysis all individuals were clustered into the same group.

**Figure S6. Repetitive elements near sdY and those causing false-positive associations with sex.** A) Screenshot from IGV of contig NW_026282589.1 (NCBI accession). Top: Each row represents an individual. Each column represents a SNP (white: missing, dark blue: homozygous, light blue: heterozygous, and green: homozygous for the alternative allele). Exons of sdY are highlighted at the top. Bottom: Resequenced genome read alignments from a diploid male and a doubled haploid female. The reads from the female only align at a putative repetitive element. B) A chi-square analysis of a SNP in the putative repetitive near sdY. The sdY contig haplotype is shown for comparison. C) IGV screenshots of SNP genotypes (same colour scheme as in A) in putative repetitive elements (labelled on the left) and alignment results to the genome assembly on the right. All regions (41 bp) aligned to multiple locations within the genome assembly. The first example is from B, the second and third are SNPs tied for the lowest p-value (these variants are labelled) from the genome-wide association analysis (LG 3 and LG 26).

**File S1. Resequenced genome sample information.** The *Biosamples* tab of this spreadsheet file contains metadata about the samples and includes the NCBI BioProject accession. The *SRA* tab has the SRA accession information. The *GPS* tab has the latitude and longitude values used for the map. The *FamilyFile* and *Covariate* tabs contains the information used in the genome-wide association analysis.

**File S2. Collapsed genome regions.** The *CollapsedRegions* tab of this spreadsheet file contains the start and end positions of the collapsed regions. The *OtherHomeologousRegions* tab has the start and end positions of homeologous regions. The *Coverage* tab has the coverage of 1 Mbp windows for each window in the genome assembly. The *GO* tabs contains the enriched GO terms from the collapsed regions of the genome assembly >3 Mbp (also the reduced REVIGO set). The *NCBI Metrics* tab has information on the number of scaffolds per chromosome and the estimated percent highly homeologous or collapsed regions per chromosome (consistent with the Circos plots).

**File S3. SnpEff impact annotations.** The *SummaryInformation* tab of this spreadsheet file contains counts and diagrams of the number of genes with the various impact SNPs. The *High* tab has the list of genes with at least one high impact SNP. The *GOHigh* tab has the results from an analysis of enriched GO terms of genes with at least one high impact SNP (none significant after adjusted for multiple tests). The *Moderate (Count)* tab contains the list of genes with at least one moderate impact SNP. The *GOModerate (Count)* tab contains the results from an analysis of enriched GO terms of genes with at least 13 moderate impact SNPs. The *GO1901223 (Count)* tab lists the genes for the enriched GO terms GO:1901223 and GO:0045341. The other tabs contain similar information for the density analysis and for genes with low impact variants.

References:

Kapustin, Y., A. Souvorov, T. Tatusova, and D. Lipman, 2008 Splign: algorithms for computing spliced alignments with identification of paralogs. Biol Direct 3: 20.

Kolde, R., 2019 pheatmap: Pretty Heatmaps.
