# Supplementary figures and images for: Insights from a chum salmon (Oncorhynchus keta) genome assembly regarding whole-genome duplication and nucleotide variation influencing gene function
Source: G3 (Bethesda). 2023 Jun 9;13(8):jkad127. doi: 10.1093/g3journal/jkad127 (PMC10411575; doi:10.1093/g3journal/jkad127)

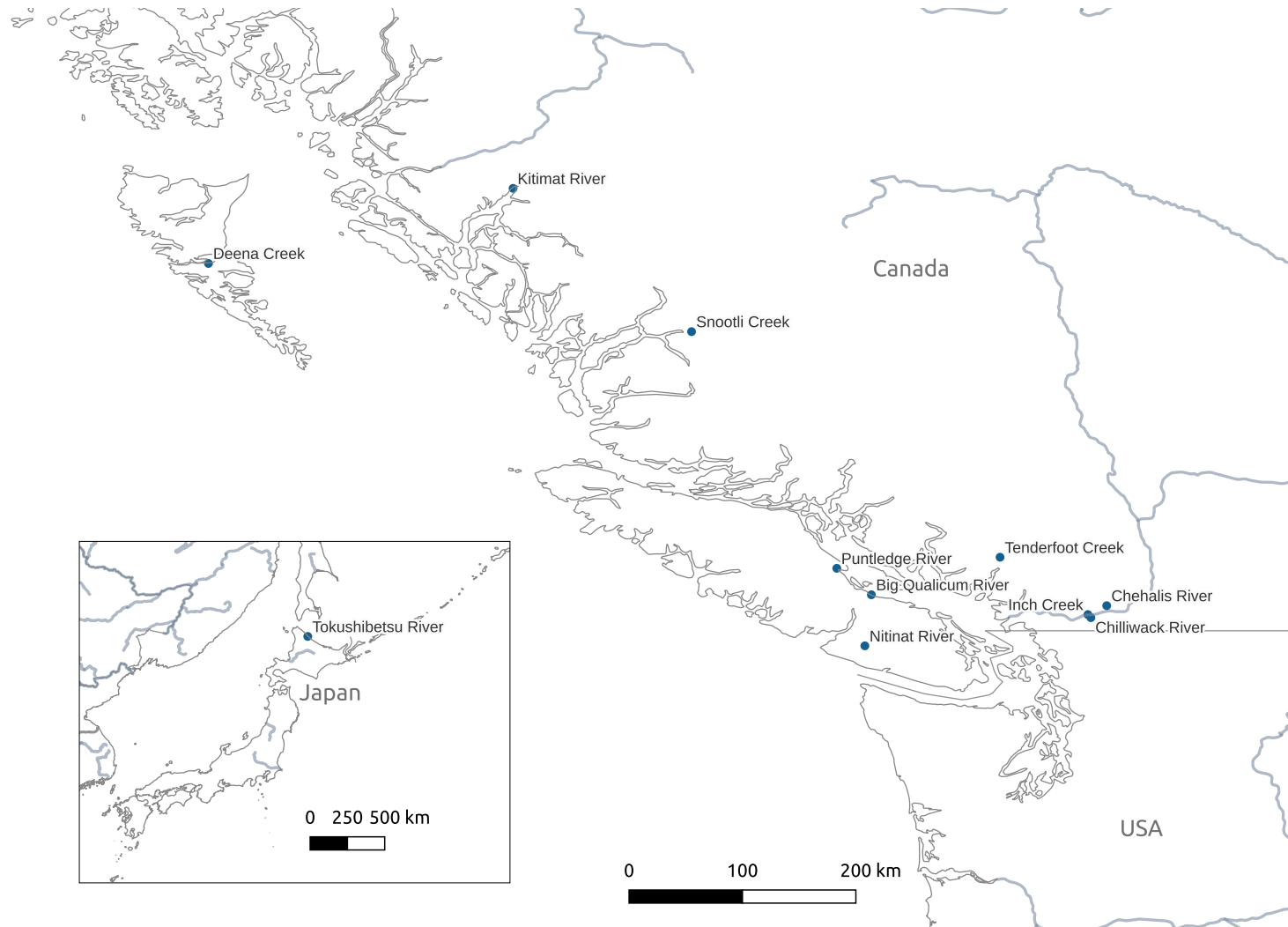

Supplement: jkad127_Supplementary_Data [file jkad127_supplementary_data.zip › Figure_S1_G3-2023-404248.pdf]

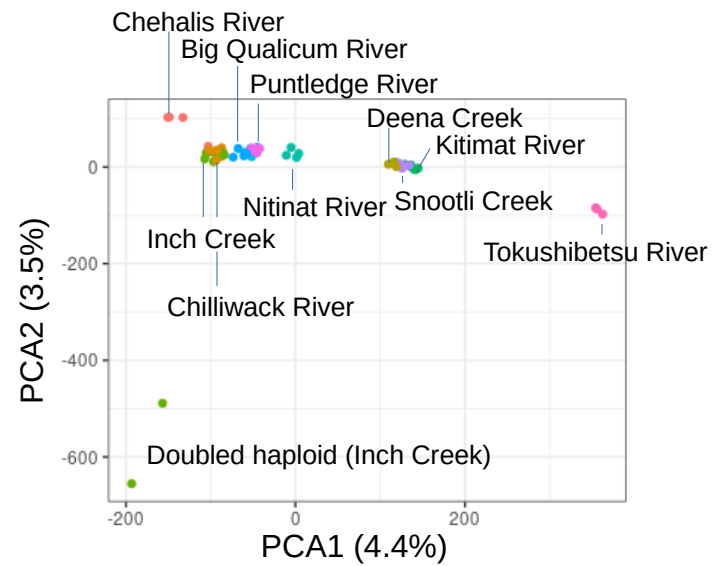

Supplement: jkad127_Supplementary_Data [file jkad127_supplementary_data.zip › Figure_S2_G3-2023-404248.pdf]

A

NW\_026282589.1

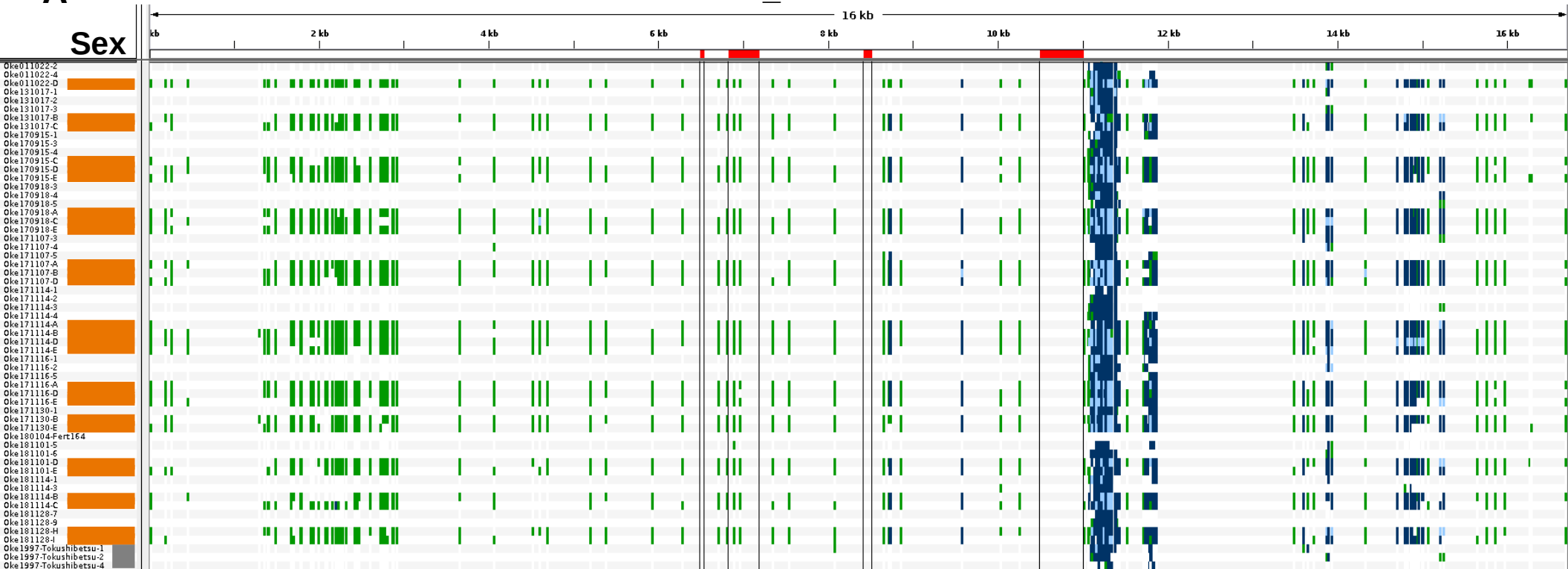**Genotypes**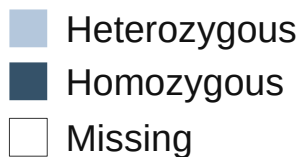

■ Homozygous  
alternative  
allele

**Sex**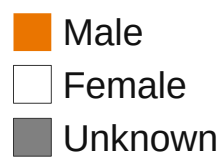

B

XM\_046332200.1 (pink salmon sdY mRNA) vs. NW\_026282589.1

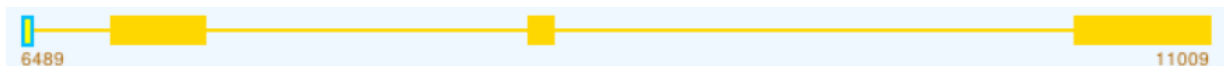

Supplement: jkad127_Supplementary_Data [file jkad127_supplementary_data.zip › Figure_S3_G3-2023-404248.pdf]

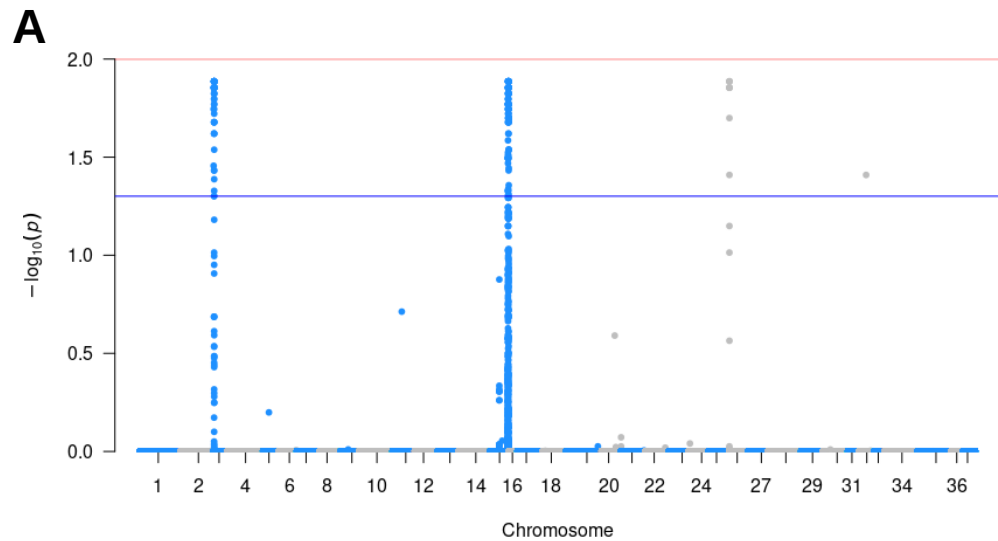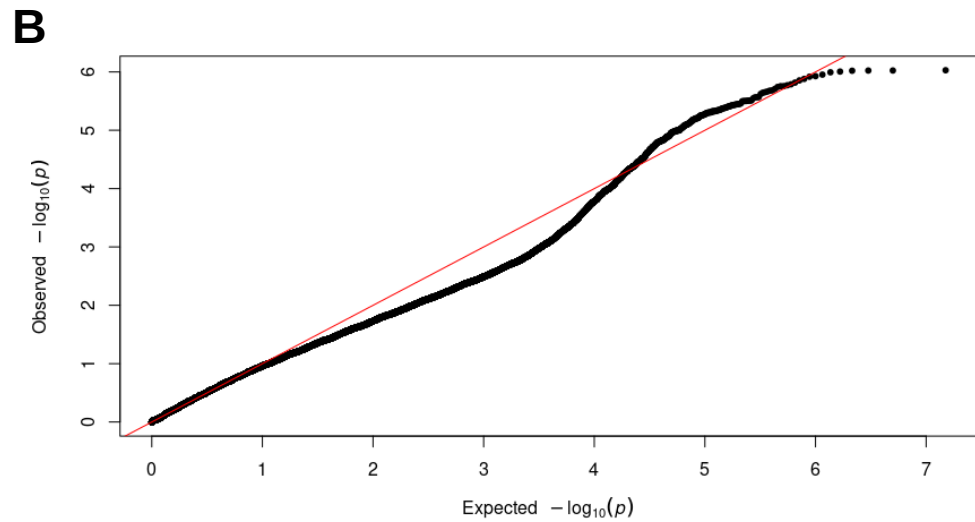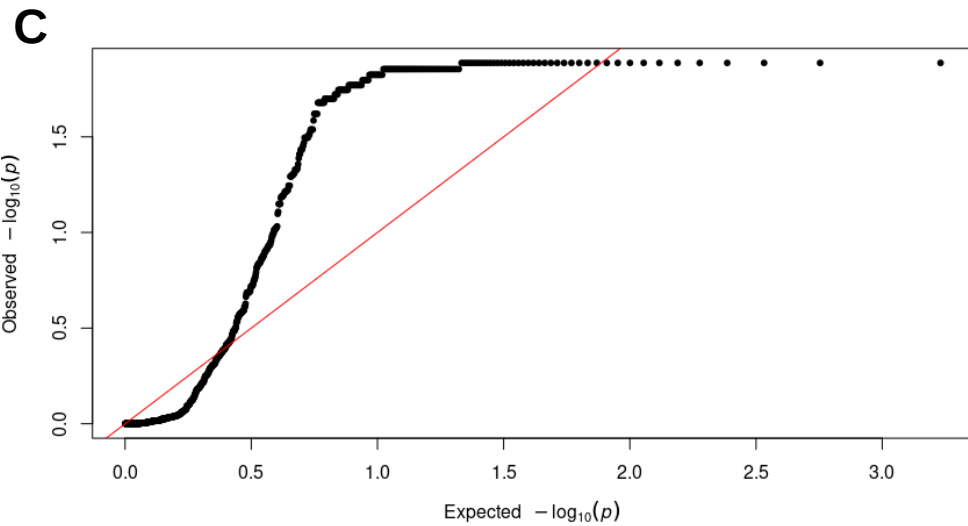

Supplement: jkad127_Supplementary_Data [file jkad127_supplementary_data.zip › Figure_S4_G3-2023-404248.pdf]

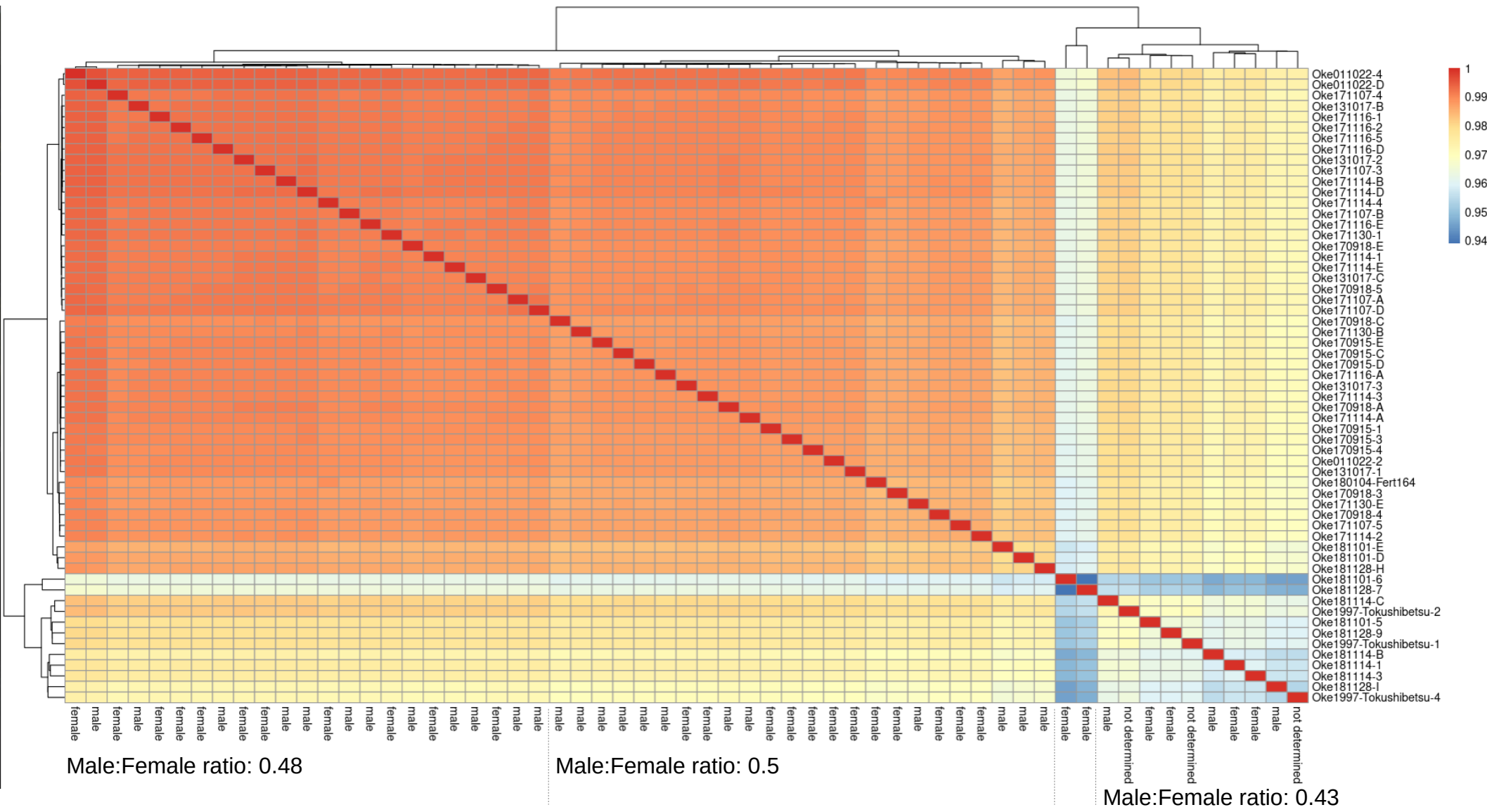

Supplement: jkad127_Supplementary_Data [file jkad127_supplementary_data.zip › Figure_S5_G3-2023-404248.pdf]
